# Reliable reconstruction of the complex high-location bile duct injury: a novel hepaticojejunostomy

**DOI:** 10.1186/s12893-019-0642-1

**Published:** 2019-11-21

**Authors:** Yuxin Zhang, Jianping Zhao, Songshan Chai, Zhanguo Zhang, Lei Zhang, Wanguang Zhang

**Affiliations:** Hepatic Surgery Center, Tongji Hospital, Tongji Medical College, Huazhong University of Science and Technology, No. 1095 Jiefang Avenue, Wuhan, 430030 Hubei province China

**Keywords:** Bile duct injury, Hepaticojejunostomy technique

## Abstract

**Background:**

This study aimed to propose a novel surgical reconstruction technique for complex high-location bile duct injury (CHBDI).

**Methods:**

There were eight patients with CHBDI underwent the novel hepaticojejunostomy between Feb 2015 and Feb 2017. Seven patients underwent a primary operation and found CHBDI postoperatively in the inferior hospitals referred to our center. And four of them had received hepaticojejunostomy, but the results were not satisfying. One patient (No.8) with radiographically diagnosed hilar cholangiocarcinoma came to our center for surgical treatment and underwent the novel hepaticojejunostomy technique because CHBDI was found in operation. Perioperative and follow-up data of these patients were retrospectively reviewed.

**Results:**

The mean age was 47.6 ± 10.7 years, and there was four female. The mean range of time between the injury and the repair operation in our center was 6.3 ± 4.8 months. All repair operations using the novel hepaticojejunostomy technique in our center were successfully performed. No postoperative complications, including biliary fistula, restenosis, peritonitis, and postoperative cholangitis was observed. Besides, no evidence of biliary stenosis or biliary complications happened during the follow-up (median 28 months).

**Conclusions:**

The novel hepaticojejunostomy is a reliable and convenient technique for surgical repair of multiple biliary ductal openings like CHBDI.

## Background

Bile duct injury (BDI) is a serious complication of hepatobiliary surgery. It can be a catastrophe, leading to high mortality, considerable financial problems, reduced quality of life, and high subsequent litigation rate [1–4]. Moreover, with the widespread adoption of the laparoscopic technique, the incidence of BDI increased sharply up to 0.5–0.8% [5–7]. High-quality reconstruction of the injured bile duct has become a knotty problem for hepatobiliary surgeons.

**Fig. 1:**
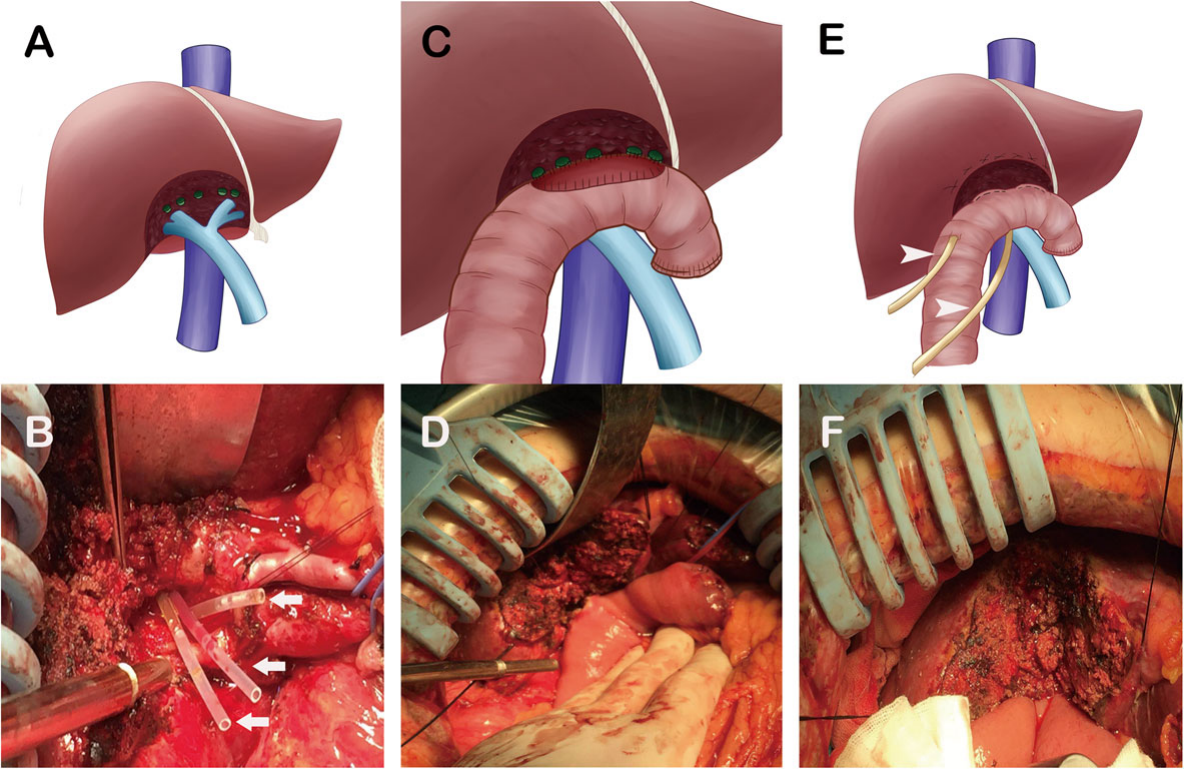
The novel hepaticojejunostomy technique: (**a**, **b**) Multiple intrahepatic bile duct openings were left after partial resection of segment IV b base and partial segment V, and the indicator tubes were placed into the proximal bile duct stumps (arrow); (**c**, **d**) the posterior wall of reconstruction used mucosa-to-mucosa anastomosis between the posterior wall of the biliary ducts and the posterior wall of the intestine; (**e**, **f**) the anterior wall of anastomosis was constituted by the suture of the anterior wall of the intestine and liver transection plane, which closely adhered to the anterior of the bile duct. A rubber decompression tube and a drainage tube were routinely placed in the intestine near the anastomotic stoma and around the anastomosis, respectively (arrowhead)

The purpose of the surgical reconstruction of the injured bile duct is to restore bile duct continuity. The strict application of healthy (i.e., non-ischemic, non-inflammation and non-scarred) bile ducts for tension-free anastomosis is the fundamental principle of high-quality bile duct reconstruction, which must drain all liver segments [8, 9]. Roux-en-Y hepaticojejunostomy (HJ) currently is recognized as the best treatment option for most major BDI to provide excellent long-term outcomes [10]. However, complex high-location bile duct injury (CHBDI) that is defined as a bile duct injury at the conjunction of the left and right hepatic duct or higher plane (classified as Strasberg classification type E4) in this study is one of the most feared types of injury [9, 11]. For the surgical treatment of CHBDI, the dissection of the hepatoduodenal ligament is inevitable to expose the injured bile duct satisfactorily. To achieve this, surgeons have to lower the hilar plate and remove partial liver parenchyma. The resection of segment IV b base and partial segment V is conducive to exposure and identify the injured bile duct for subsequent high-quality bilioenteric anastomosis [12, 13]. Unfortunately, this operation inevitably leaves multiple intrahepatic bile duct openings (Fig. 1a and b), which is very difficult for traditional end-to-end or side-to-side anastomosis [11]. It is worth mentioning that the openings of the small bile duct are the branches of left and right hepatobiliary duct and the opening of the caudate lobe bile duct.

In such intractable situations, there is not yet a unified and effective surgical approach to solve this problem. Therefore, we proposed a novel HJ technique to address the anastomosis problem of CHBDI. In our previous study, we have tested this new HJ technique for multiple biliary duct openings in pigs and got excellent results [11]. We herein presented the results of a preliminary clinical study applying this technique in 8 CHBDI patients.

## Methods

### Patient selection

From Feb 2015 to Apr 2017, eight patients with CHBDI underwent the novel HJ by one surgeon (WGZ) at the Hepatic Surgery Center, Tongji Hospital, Tongji Medical College, Huazhong University of Science and Technology (HUST). Among them, seven patients who underwent primary laparoscopic cholecystectomy (LC) and found CHBDI postoperatively in the inferior hospitals referred to our center. And four of them had received HJ (the specific way of the anastomosis is unknown), but the results were not satisfying. One patient (No.8) with radiographically diagnosed hilar cholangiocarcinoma came to our center for surgical treatment. Because we found hilar and intrahepatic bile ducts dilatation with mucus-like substance. Intraoperative pathological diagnosis is highly suspicious mucin-producing intrahepatic biliary papillomatosis (MPIBP), which was confirmed after surgery. Trying to remove the injured bile duct in principle and taking into account the remaining liver volume, we performed palliative bile duct resection and the novel HJ. All patients underwent informed consent for surgery.

### Pre-operative preparation

Preoperatively, routine examination such as complete blood count, blood biochemical parameters, coagulation function, and cardiopulmonary function was tested. Comprehensive imaging of the bile duct condition should be obtained before the repair of IBDI. Percutaneous transhepatic cholangiography (PTC), endoscopic retrograde cholangiopancreatography (ERCP), computed tomography (CT), fistulography, or magnetic resonance cholangiopancreatography (MRCP) were performed to make a comprehensive evaluation.

### Surgical technique

A left lateral decubitus position is routinely used. Based on the location of the injures, a J incision on the right-side of the upper abdomen or bilateral subcostal laparotomy was performed. The delicate hilar dissection to expose the inferior of the segment IVb was required. The small bridge of liver tissue connecting the quadrate lobe (segment IVb) with the left lateral lobe was sectioned, and the gallbladder plate or umbilical plate was liberated to ensure exposure, if necessary.

### Exposure of the injured bile duct

Since the loss of bifurcation and high location of the injury, healthy bile duct might be embedded in the liver parenchyma, which could not be exposed by lowering the liver hilar plate and dissecting hepatoduodenal ligament, the resection of the partial segment IV b base and segment V was needed to improve exposure. Liver resection would inevitably lead to several small bile duct openings. However, the novel HJ technique could excellently address this kind of anastomosis problem. We usually expose the left and right hepatobiliary ducts and the caudate lobe bile duct as healthy bile ducts for anastomosis.

### Construction of anastomosis

The duodenum, jejunum and ileum fistula were carefully examined prior to the anastomosis. A Roux-en-Y loop of jejunum was prepared by dividing the proximal jejunum 20 to 40 cm below the Treitz ligament. The distal transected end was closed, and the jejunum was brought up to the portahepatis region through a retrocolic route. The gastrointestinal tract was reconstructed using an end-to-side jejunojejunostomy. Any missed open duct could lead to a late occurrence of cholangitis, which even needed reoperation to repair. Therefore, at the transected cut plane, all small bile duct openings were meticulously explored by using the preoperative imaging studies or PTCD as an intraoperative roadmap. Then, the indicator tubes were placed into the proximal bile duct and would be removed after anastomosis.

In the novel HJ, the posterior wall of reconstruction was performed by mucosa-to-mucosa anastomosis between the posterior wall of the biliary ducts and the posterior wall of the intestine (Fig. 1c and d). The anterior wall of anastomosis was sutured between the anterior wall of the intestine and liver transection plane, which closely adhered to the anterior of the bile duct (Fig. 1e and f.). The anterior of the bile duct was unsutured. Bile duct or hilar plate was meticulously sutured with 5–0 or 6–0 absorbable suture. Rinsing the surgical field with saline until the fluid is clear. A rubber decompression tube and a drainage tube were routinely placed in the intestine near the anastomotic stoma and around the anastomosis, respectively, to prevent and to monitor anastomotic fistula (Fig. 1e).

## Results

Totally eight patients who underwent the novel suture technique because of CHBDI from Feb 2015 to Feb 2017 at our center were enrolled in this preliminary study. General data were shown in Table 1. The mean age was 47.6 ± 10.7 years, and there were four females. All cases were bile duct injury at the conjunction of the left and right hepatic duct or higher plane and classified as Strasberg E4. The operative time and the intraoperative blood loss were 231.5 ± 77.0 min and 76.3 ± 60.2 ml, respectively, which had no statistically significant difference with our previous classical HJ (data not shown). The mean range of time between the injury and the repair operation in our center was 6.3 ± 4.8 months. All repair operations using the novel HJ technique in our center were successfully performed.

**Table 1:** The general data of the patients with CHBDI who underwent repair operation using the novel cholecystectomy technique

Patient	Gender	Age	Primary operation	Operative time	Intraoperative blood loss	Type of injury^a^	Range of time^b^	Postoperative complications	Follow-up
1	Female	43	LC	170 min	20 ml	E4	3 months	No	19mon
2	Male	48	LC + hepaticojejunostomy	223 min	80 ml	E4	12 months	No	21mon
3	Male	53	LC	169 min	50 ml	E4	4 months	No	23mon
4	Female	32	LC + hepaticojejunostomy	263 min	100 ml	E4	8 months	No	29mon
5	Female	38	LC + hepaticojejunostomy	189 min	10 ml	E4	6 months	No	31mon
6	Male	56	LC	236 min	100 ml	E4	3 months	No	35mon
7	Female	45	LC + hepaticojejunostomy	198 min	50 ml	E4	14 months	No	39mon
8	Male	66	Exploratory laparotomy	404 min	200 ml	E4	Acute	No	27mon

*CHBDI* complex high-location bile duct injury, *LC* laparoscopic cholecystectomy

^a^, Strasberg classification

^b^, the range of time between the injury and the repair operation in our center

**Fig. 2:**
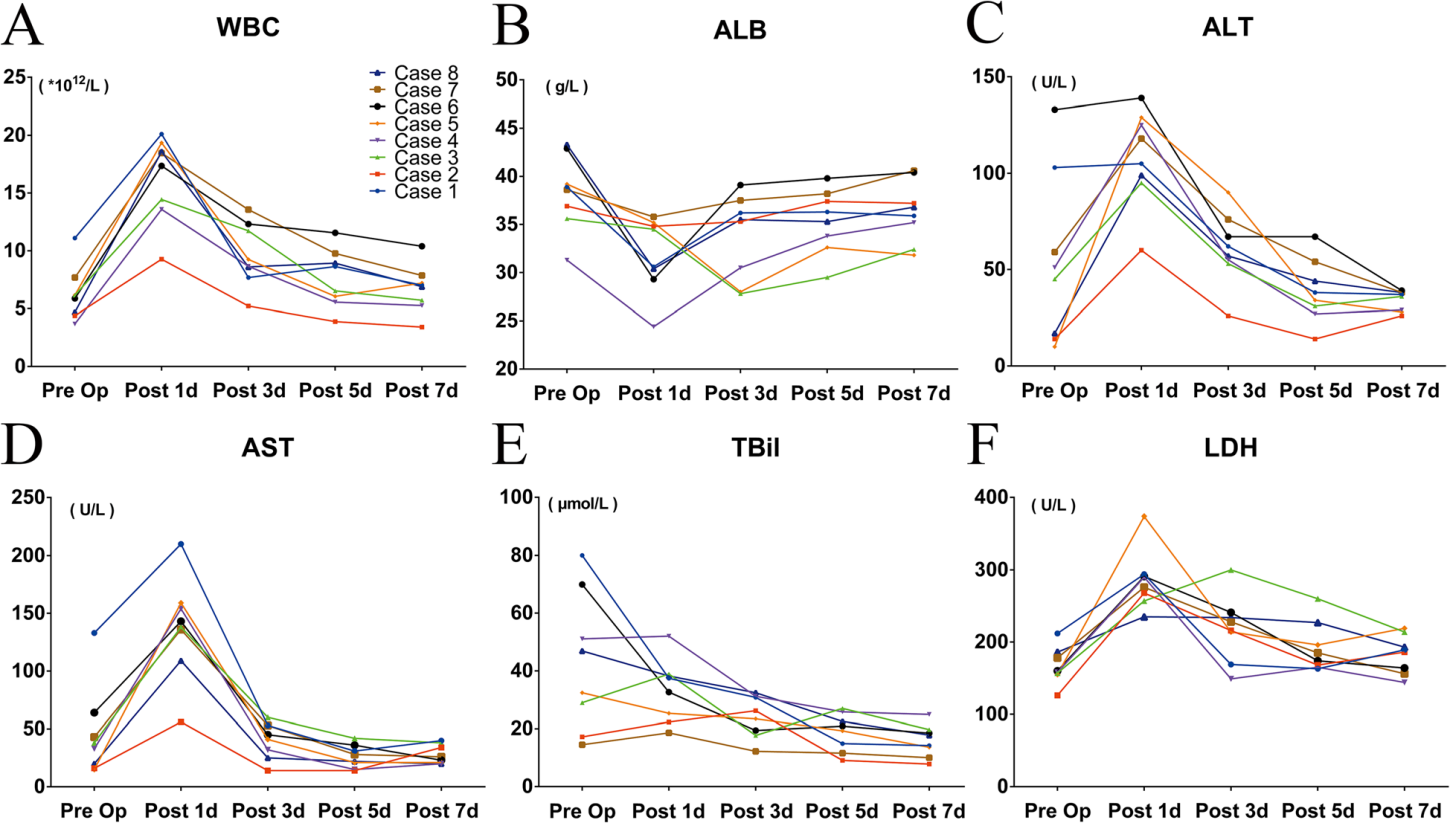
Dynamic changes of blood test Pre- and Post-operation. WBC: white blood cell; ALB: Albumin; ALT: alanine transaminase; AST: aspartate transaminase; TBil: Total bilirubin; LDH: lactate dehydrogenase

Figure. 2 presented the dynamic changes of the blood tests (including white blood cell count (WBC), serum albumin (ALB), alanine transaminase (ALT), aspartate transaminase (AST), total bilirubin (TBil), lactate dehydrogenase (LDH)) before and after the operation. The results showed that the levels of the WBC, ALT, AST, and LDH in all the eight patients increased on the first postoperative day. Thereafter, the levels of WBC, ALT, and AST began to decrease on the third postoperative day and restored to the normal levels on the fifth postoperative day. The levels of AST had restored to the normal level on the third postoperative day. Meanwhile, the levels of ALB in all patients and the levels of TBil in half of the patients decreased on the first postoperative day. On the third postoperative day, the levels of ALB were elevated, and the levels of the TBil came to decline in most of the patients, and both of them restored to the normal levels on the fifth postoperative day. The results of the blood examinations after a long-term follow up were shown in Table 2. The ALT, AST, and LDH were 22 ± 5.3 U/L, 28.4 ± 5.8 U/L, and 161.1 ± 22.7 U/L, respectively.

**Table 2:** Follow up of long-term blood examination in all eight cases

Patient	ALT (U/L)	AST (U/L)	LDH (U/L)	Postoperative time
1	19	22	134	19mon
2	16	32	168	21mon
3	23	28	156	23mon
4	19	26	183	29mon
5	25	36	178	31mon
6	17	20	132	35mon
7	25	28	146	39mon
8	32	35	192	27mon

*ALT* alanine transaminase, *AST* aspartate transaminase, *LDH* lactate dehydrogenase

**Fig. 3:**
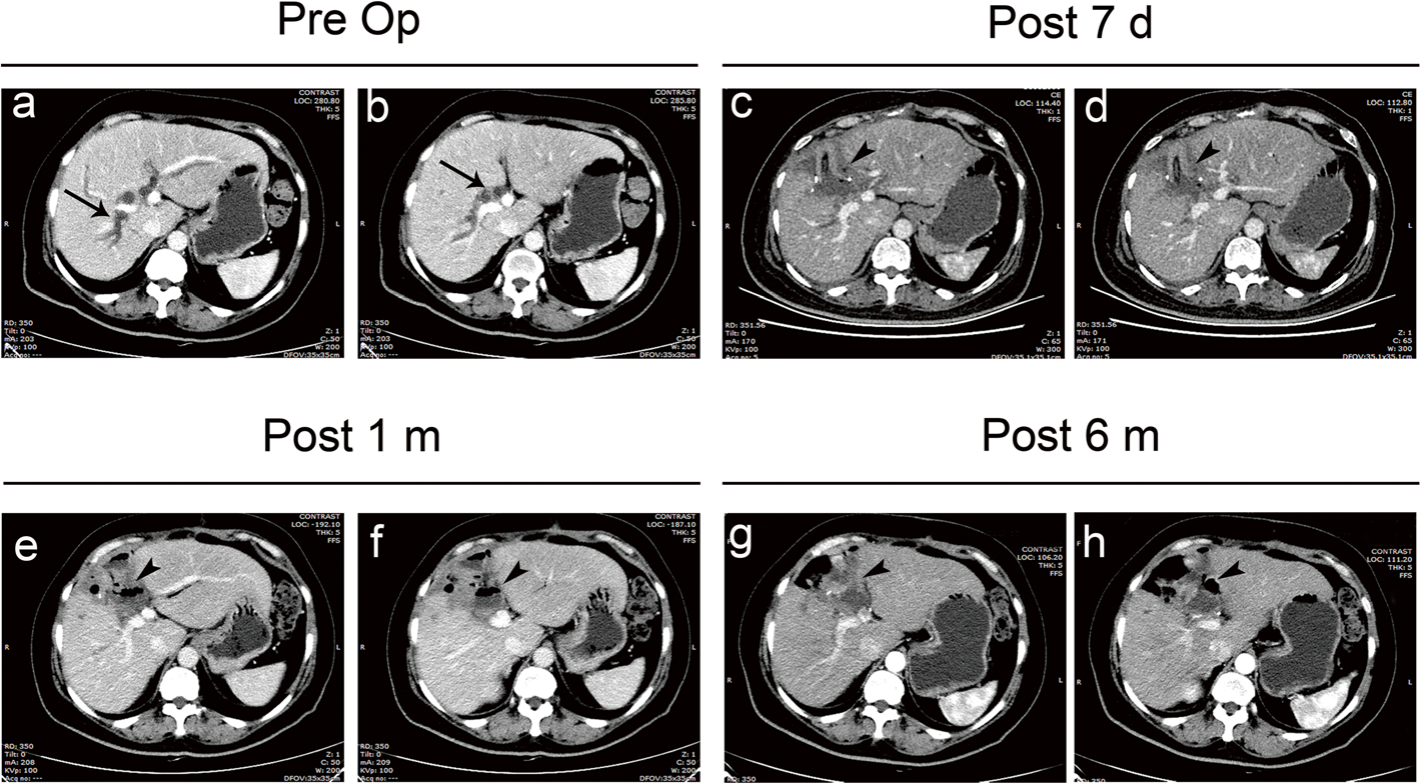
The CT scans of the No.1 patient before and after the operation. (**a**, **b**) The dilated bile ducts before surgery (arrow); (**c**, **d**, **e**, **f**, **g**, **h**) The dilated bile ducts had restored to normal after the operation, and the anastomotic stoma healed well without stenosis (arrowhead). Pre OP: pre-operation; Post 7 d: post-operation 7th day; Post 1mon: post-operation 1st month; Post 6mon: post-operation 6th month

In our center, the CT scan was not routinely performed after the operation unless the patient presents with fever and jaundice symptoms. However, we took the CT scan in the No. 1 patient in the 7th postoperative day, the first month, and the sixth month after the operation as the patient’s requirement. The CT images were presented in Fig. 3. The bile ducts were obviously dilated before the operation and were almost restored to normal after the operation. Besides, the anastomotic stoma healed well at the resection site of partial hepatic parenchyma.

No postoperative complication was observed, including biliary fistula, restenosis, peritonitis, and postoperative cholangitis. Besides, no evidence of biliary stenosis or biliary complications happened during the follow-up (median 28 months).

## Discussion

BDI, especially the CHBDI is still a challenging issue for hepatobiliary surgeons, even for those experienced surgeons. According to the type of injury, percutaneous, endoscopic, and surgical treatments could be used to cope with the management of BDI [14]. In recent decades, surgical interventions with Roux-en-Y HJ has been regarded as the best treatment option for the BDI [10, 15]. However, for CHBDI, resection of segment IV b base and partial segment V is recommended to achieve adequate boundary of healthy (non-ischemic, non-inflammation and non-scarred) ducts and improve the surgical space, which therefore inevitably leave multiple small intrahepatic bile duct openings [13, 14, 16, 17]. In such a situation, the traditional HJ anastomosing the hepatic duct to the jejunum using mucosa-to-mucosa anastomosis is difficult for small bile ducts and limits the operative space [11, 18]. The Hepp-Couninaud HJ (side-to-side anastomosis) technique reported by Hepp was once considered as an ideal method for Strasberg E1-E3 injuries [13, 19]. Thereafter, Winslow and colleagues adopted this side-to-side anastomosis technique in some complicated E4 or E5 bile duct injuries and achieved excellent anastomotic function [20]. However, there is no mention of segmental branches anastomosis to solve the multiple biliary duct openings and the difficulty of this side-to-side anastomosis increases with the diameter of the bile duct decreases. Therefore, it is unrealistic to perform this technique in CHBDI. Ren proposed an HJ technique (morioplasty with round ligament) for high-location BDI, and Hwang reported a cluster HJ to deal with severely hilar bile ducts injuries [21, 22]. Both anastomosis procedures performed by suturing the round ligament or a jejunal loop to the liver parenchyma around the transected hepatic ducts, which are similar to the Kasai procedure [23]. Compared with the preceding methods, the innovation of our novel HJ technique is that the posterior wall of the bile ducts is sutured to the posterior wall of jejunum with the mucosa-to-mucosa anastomosis, while the anterior wall of the jejunum is sutured to the liver transection plane, which closely adhered to the anterior wall of the bile ducts. And the anterior of the bile duct is unsutured. The operative space is large, and the operative field is clear, the operation, therefore, is relatively easier. Most important of all is that no postoperative biliary complication was observed, including biliary fistula, restenosis, peritonitis, and postoperative cholangitis after the operation and during the follow-up period in the present study. Moreover, our previous study adopting this technique in pig have revealed histologically that the anterior wall of the anastomotic stoma was healed later than the posterior wall and result in the anterior wall of hepatic stoma everted, which leading to a satisfactorily lower degree of stenosis than the traditional anastomosis [11].

The use of the stent in the bile duct stump for supporting the bile ducts and preventing biliary strictures is controversial [14, 24]. In the novel HJ technique, the use of a stent is not needed or recommended. The best timing to repair the BDI has not been defined. Iannelli and colleagues suggested that the best results of repair of BDI could be expected by experienced hepatobiliary surgeons using the Roux-en-Y HJ beyond 45 days after the occurrence of injury [25]. However, Stewart and colleagues found that the factors influencing the surgical outcomes of repair of BDI were the eradication of intra-abdominal infection, complete preoperative cholangiography, use of correct surgical technique, and repair by a biliary surgeon, while the timing of repair was unimportant [26]. In the present study, the mean range of time between the injury and the repair operation in our center was 7.1 months. It is worth noting that there was one repair of CHBDI performed immediately when the injury was found in the primary operation. Our experience recommends that the repair should be considered when the injury is found no matter it is found during or after the primary operation.

The present study had several limitations. The first limitation was its retrospective nature. Secondly, the sample size was small. As a preliminary clinical trial, only 8 cases were enrolled in the present study. Thus, a prospectively multicenter study with a larger sample size was needed to further evaluate the safety and effectiveness of the novel hepaticojejunostomy. Thirdly, only one of the eight patients had token the CT scans during the follow-up, which limited us to comprehensively access the long-term outcomes of the novel hepaticojejunostomy, although the blood examination during the follow-up showed no cholangitis occurred and the liver function was normal.

## Conclusions

The novel HJ is a reliable and convenient technique for surgical repair of multiple biliary ductal openings like CHBDI, and we recommend it is best to be conducted by experienced hepatobiliary surgeons.

## Data Availability

The datasets generated and/or analyzed during the current study are not publicly available due to protecting individual patient privacy but are available from the corresponding author on reasonable request.

## Abbreviations

BDI: Bile duct injury
CHBDI: Complex high-location bile duct injury
HJ: Hepaticojejunostomy
MPIBP: Mucin-producing intrahepatic biliary papillomatosis
